# Optimized prediction of diabetes complications using ensemble learning with Bayesian optimization: a cost-efficient laboratory-based approach

**DOI:** 10.3389/fendo.2025.1593068

**Published:** 2025-06-20

**Authors:** Dapeng Yan, Xiaohan Li, Yifan Wang, Zhikuang Cai

**Affiliations:** ^1^ College of Integrated Circuit Science and Engineering, Nanjing University of Posts and Telecommunications, Nanjing, China; ^2^ Laboratory Medicine Center, The Second Affiliated Hospital, Nanjing Medical University, Nanjing, China; ^3^ Department of Hematology, The Affiliated Huaian No.1 People’s Hospital of Nanjing Medical University, Huai’an, China

**Keywords:** diabetes complications, predictive modeling, machine learning, Bayesian optimization, cost-efficient diagnosis, clinical laboratory indicators

## Abstract

**Background and objective:**

The increasing global prevalence of diabetes has led to a surge in complications, significantly burdening healthcare systems and affecting patient quality of life. Early prediction of these complications is critical for timely intervention, yet existing models often rely heavily on clinical indicators while underutilizing fundamental laboratory test parameters. This study aims to bridge this gap by leveraging the 12 most frequently tested laboratory indicators in diabetic patients to develop an optimized predictive model for diabetes complications.

**Methods:**

A comprehensive dataset was established through meticulous data collection from a high-volume tertiary hospital, followed by extensive data cleaning and classification. Various machine learning classifiers, including Random Forest, XGBoost, Support Vector Machine (SVM), and Multilayer Perceptron (MLP), were trained on this dataset to evaluate their predictive performance. We further introduced an ensemble learning model with Bayesian optimization to enhance accuracy and cost-efficiency. Additionally, feature importance analysis was conducted to refine the model by reducing testing costs while maintaining high predictive accuracy.

**Results:**

Our ensemble model with Bayesian optimization demonstrated superior performance, achieving over 90% accuracy in predicting various diabetic complications, with an outstanding 98.50% accuracy and 99.76% AUC for diabetic nephropathy. Feature correlation analysis enabled a refined model that not only improved predictive accuracy but also reduced overall medical costs by 2.5% through strategic feature elimination.

**Conclusions:**

This study makes three key contributions: (1) Development of a high-quality dataset based on fundamental laboratory indicators, (2) Creation of a highly accurate predictive model using ensemble learning and Bayesian optimization, particularly excelling in diabetic nephropathy prediction, and (3) Implementation of a cost-efficient diagnostic approach that reduces testing expenses without compromising accuracy. The proposed model provides a strong foundation for future research and practical clinical applications, demonstrating the potential of integrating machine learning with cost-conscious medical testing.

## 1 Introduction

Diabetes mellitus is more than a chronic disease—it is a global health crisis that threatens millions of lives and places immense pressure on healthcare systems. Characterized by persistent hyperglycemia due to defects in insulin secretion, insulin action, or both, diabetes is classified into Type 1 diabetes (T1D) and Type 2 diabetes (T2D). T1D, often diagnosed in childhood or adolescence, results from the autoimmune destruction of pancreatic beta cells, while T2D, which accounts for over 90% of diabetes cases, is primarily driven by insulin resistance and metabolic dysfunction (1, 2).

The most devastating aspect of diabetes is not the disease itself but its complications. Hyperglycemia silently wreaks havoc on multiple organ systems, leading to both acute and chronic complications. Acute events such as diabetic ketoacidosis and hyperosmolar hyperglycemic state demand immediate medical intervention and can be fatal if left untreated (3, 4). However, the real danger lies in the chronic complications, which develop gradually over time, often remaining undetected until irreversible damage has occurred. These include cardiovascular disease, kidney failure, blindness, and neuropathy, all of which severely impact patients’ quality of life and contribute to premature mortality (5, 6).

The scale of this crisis is staggering. According to the International Diabetes Federation (IDF), 537 million adults were living with diabetes in 2021, and this number is expected to rise to 700 million by 2045 (7, 8). In many high-income countries, diabetes prevalence has already exceeded 10%, overloading healthcare infrastructure and driving up medical costs (9). Meanwhile, rapid urbanization, sedentary lifestyles, and dietary changes are fueling an alarming surge in T2D cases across developing nations (10). Given the inevitable increase in diabetes-related complications, the ability to predict these complications early and accurately is becoming an urgent priority.

Despite significant advancements in diabetes management, the early detection of complications remains a critical challenge. Many of these complications progress asymptomatically, making timely intervention difficult. The accuracy of manual diagnosis varies depending on the type of complication and clinical setting, ranging from 70% to 90%. For instance, diabetic retinopathy is typically diagnosed through fundoscopic examination and retinal imaging, with diagnostic accuracy depending on physician experience (11, 12). Diabetic nephropathy is commonly assessed using serum creatinine, urinary albumin, and microalbuminuria measurements, with studies suggesting that pentoxifylline may offer nephroprotective effects (2, 3). Cardiovascular complications, which remain the leading cause of mortality among diabetic patients, are often evaluated based on HbA1c levels, lipid profiles, and blood pressure, yet fluctuations in HbA1c have been strongly linked to an increased risk of cardiovascular events (3, 5, 6). Similarly, diabetic neuropathy is diagnosed through nerve conduction studies and neurophysiological tests, with emerging research identifying decreased genomic DNA methylation as a potential biomarker for neuropathy risk in T2D patients (1, 13).

Given the limitations of traditional diagnostic approaches, there is a pressing need for more reliable, data driven methods to predict diabetic complications before they manifest clinically. The emergence of machine learning (ML) has revolutionized medical research, enabling the identification of complex, non-linear patterns in vast datasets that would otherwise be undetectable through conventional statistical methods (14, 15). Recent machine-learning (ML) techniques—including logistic regression, decision tree, random forest, and support vector machine—have demonstrated strong predictive performance for a variety of diabetic complications, often achieving AUCs above 0.85 in external validations (16, 17). However, despite this promise, existing models typically (1) draw on only a limited set of clinical indicators and (2) target one complication at a time.

• Limited diversity of predictive features

Most models incorporate HbA1c, blood pressure, and basic lipid panels, but omit routinely collected indicators such as uric acid, creatinine, and full lipid subfractions (18, 19). Recent work (20, 21) shows that features such as blood glucose, creatinine, and uric acid significantly improve the prediction of retinopathy and nephropathy when added to simple predictors.

• Focus on a single complication

Diabetic retinopathy models achieve a high negative predictive value using only minimal clinical inputs, but do not extend to nephropathy or cardiovascular disease (22, 23). Patients often develop multiple concurrent complications (e.g., nephropathy, neuropathy, and cardiovascular disease), and single-task ML frameworks clearly cannot handle this complexity (24, 25).

To bridge these gaps, this study develops a machine learning-based predictive model using the 12 most frequently tested laboratory indicators in diabetic patients. These indicators, already measured in routine clinical practice, provide an accessible and cost-effective opportunity for early complication prediction. Our hypothesis is that by integrating machine learning with these fundamental laboratory test results, we can enable earlier and more accurate complication detection, ultimately improving patient outcomes and reducing unnecessary medical expenses.

The primary contributions of this study are as follows:

1. Development of a benchmark dataset (DCD): We compile a well-curated dataset from the Second Affiliated Hospital of Nanjing Medical University, consisting of 5,000 patient records, integrating laboratory results with clinical diagnoses.
2. Implementation of an ensemble learning model with Bayesian optimization: Our model significantly outperforms traditional classifiers (SVM, XGBoost, Random Forest, MLP). Notably, our approach achieves 98.5% accuracy and 99.76% AUC in predicting diabetic nephropathy, surpassing existing methods.
3. Cost-efficient feature selection: By analyzing feature importance, we refine our model to reduce medical testing costs by 2.5%, ensuring that high diagnostic performance is maintained while eliminating unnecessary tests.

By integrating machine learning with fundamental laboratory diagnostics, this study presents a practical and scalable solution for early detection of diabetic complications. The proposed approach not only enhances predictive accuracy but also optimizes healthcare resource utilization, offering a feasible path toward more proactive and cost-effective diabetes management.

## 2 Materials and methods

### 2.1 Data collection

Hospital selection: The data used in this study were obtained from the laboratory department of the Second Affiliated Hospital of Nanjing Medical University, located in Nanjing, Jiangsu Province, China. This is a Class-A tertiary comprehensive hospital directly under the Jiangsu Provincial Health Commission, responsible for essential tasks such as medical treatment, teaching, scientific research, and public welfare. The hospital handles ^1^approximately 2.01 million outpatient and emergency visits annually, discharges about 78,000 patients, performs around 20,000 surgeries, and provides hemodialysis services to roughly 160,000 individuals each year. Thus, the hospital possesses sufficient capacity to support our experimental data collection.

Dataset description: Our dataset includes patient IDs, diabetes type, initial complication labels and 12 indicators (blood glucose, uric acid, 24-hour urine protein, HDL cholesterol, LDL cholesterol, total cholesterol, postprandial glucose, cystatin C, creatinine, urine microalbumin, urine microalbumin-tocreatinine ratio, and glycated hemoglobin). All released data have been fully de-identified to guarantee complete anonymity and irretraceability. In accordance with the “Measures for the Ethical Review of Life Sciences and Medical Research Involving Human Subjects,” ^2^this study was granted exemption from formal ethical review.

Feature selection: All twelve laboratory indicators routinely measured for diabetic patients in our department were included in the dataset. By retaining every available feature, we ensured that the model had access to the complete set of clinically relevant variables. This comprehensive inclusion avoids potential bias and allows the algorithms to determine their relative importance empirically.

Data preprocessing: Based on the selected features, we extracted the relevant indicators of all diabetes patients tested in the laboratory since October 2020, linked them according to the medical record number, and merged the data. The following two-step aggregation procedure is applied to convert multiple measurements per patient into one or more samples, according to whether the clinical diagnosis remained stable or changed over time.

Step 1: Stable diagnosis (1-to-1 mapping). When the clinical diagnosis of a patient did not change, we collapsed all the indicators into a single representative sample. Specifically, let *x_ij_* denote the *j*-th measurement of indicator *i* for that patient (*x_ij_* must hold a numerical value). We computed the arithmetic mean


$${\bar{x}}_{i}\;=\;\frac{1}{M_{i}}\sum_{j=1}^{M_{i}}x_{ij}\;,$$

where *M_i_* is the total number of measurements of indicator *i*. The vector ${\left\{{\bar{x}}_{i}\right\}}_{i=1}^{12}$ then constitutes one aggregated sample for that patient, preserving the central tendency of each indicator while eliminating intra-patient temporal redundancy.

Step 2: Evolving diagnosis (1-to-*N* mapping, *N* > 1). For patients whose diagnosis changed over time—resulting in *N* distinct diagnostic stages—we first partitioned the patient’s record into contiguous segments, each corresponding to a single diagnosis state. Within the *k*-th segment (diagnosis stage *D_k_*), we again computed the arithmetic mean of each indicator as in Step 1:


$${\bar{x}}_{i}^{\left(k\right)}\;=\;\frac{1}{M_{i}^{\left(k\right)}}\sum_{j=1}^{M_{i}^{\left(k\right)}}x_{ij}^{\left(k\right)}\;,$$

Where $M_{i}^{\left(k\right)}$ is the number of measurements of indicator *i* during stage *k*. After aggregation, we generated *N* separate samples ${\left\{{\bar{x}}_{i}^{\;\left(k\right)}\right\}}_{i=1}^{12}$ , one for each diagnostic stage *D_k_*. This procedure yields multiple summary observations per patient, each reflecting the patient’s state under a distinct diagnosis.

Using the above method, we obtained 19,043 initial data points. During the data cleaning stage, to ensure that each indicator had a corresponding value for feature extraction, we eliminated records with null test indicators, meaning patients were required to have all 12 indicators tested simultaneously. Due to the small amount of data on diabetic gastric complications (only 30 lines before merging), this data was removed after cleaning. Ultimately, we obtained 3,000 data points with complete features.

### 2.2 Tag classification

Based on the cleaned data, the clinical diagnosis results entered by each diagnosing physician were different. To unify the label data, we classified cases of essential diabetes into diabetes without complications. We did not distinguish between type 1 and type 2 diabetes complications, categorizing all into broad complication labels. The merged results are shown in **Table 1**.

**Table 1:** Label conversion.

Clinical diagnosis results	Labels
Type 2 Diabetes	Diabetes without Complications
Diabetes Type II	
Type 1 Diabetes Type I	
Diabetes	
Gestational Diabetes	
Pregnancy-induced Diabetes	
Type 2 Diabetic Peripheral Neuropathy	Diabetic Neuropathy
Diabetic Peripheral Neuropathy	
Diabetes with Neurological Complications	
Type 2 Diabetic Neurogenic Bladder	
Type 1 Diabetic Peripheral Neuropathy	
Diabetic Radiculopathy	
Type 2 Diabetic Autonomic Neuropathy	
Diabetic Retinopathy	Diabetic Eye Complications
Type 2 Diabetic Retinopathy	
Type 2 Diabetic Tractional Retinal Detachment	
Type 2 Diabetic Proliferative Hemorrhagic Retinopathy	
Diabetic Proliferative Retinopathy	
Type 2 Diabetic Proliferative Retinopathy	
Type 2 Diabetic Neovascular Glaucoma	
Diabetic Tractional Retinal Detachment	
Diabetic Neovascular Glaucoma	
Type 2 Diabetic Retinal Thickening Retinopathy	
Diabetic Foot	Diabetic Foot Complications
Type 2 Diabetic Foot	
Diabetic Gangrene	
Type 2 Diabetic Foot Gangrene	
Diabetic Foot Gangrene	
Type 2 Diabetic Lower Limb Infection	
Type 1 Diabetic Lower Limb Infection	
Type 2 Diabetic Foot Disease	
Diabetic Lower Limb Infection	
Type 2 Diabetic Foot Ulcer and Peripheral Neuropathy	
Diabetic Lower Limb Ulcer	
Type 2 Diabetic Gangrene	
Type 2 Diabetic Peripheral Vascular Disease	Diabetic Vascular Complications
Diabetic Peripheral Vascular Disease	
Type 2 Diabetic Peripheral Vascular Disease and Gangrene Type 1 Diabetic Peripheral Vascular Disease	
Diabetic Nephropathy	Diabetic Kidney Complications
Type 2 Diabetic Nephropathy	
Diabetic Nephropathy Stage III	
Diabetes with Renal Complications	
Diabetic Nephropathy Stage IV	
Diabetic Nephropathy Stage II	
Diabetic Nephropathy Stage I	
Type 2 Diabetic Nephropathy Stage II Diabetic Nephropathy Stage V	

Following the label merging process, we addressed the class imbalance in the dataset to ensure equitable representation across all categories. As shown in **Table 2**, the original dataset exhibited significant imbalance, with the largest category, “Diabetes without complications,” comprising 7556 samples. Given the impracticality of increasing the sample size of the minority classes to several thousand, we opted to constrain the sample size of each category to 500. To achieve this balanced distribution, we employed the SMOTE algorithm (26), which facilitated both oversampling of the minority classes and undersampling of the majority class through a nearest-neighbor approach. As a result, we obtained a balanced dataset with 500 samples per category across 6 labels, yielding a total of 500 (balanced sample size) × 6 (number of labels) = 3000 data points. This balanced dataset, referred to as the DCD dataset, was used for subsequent analyses.

**Table 2:** Sample size and proportion relative to largest category for various diabetes complications.

Category	Sample size	Proportion relative to largest category
Diabetes without complications	7556	1.0000
Diabetic ocular complications	344	0.0455
Diabetic neurological complications	331	0.0438
Diabetic foot complications	285	0.0377
Diabetic vascular complications	264	0.0349
Diabetic nephropathy	253	0.0335

### 2.3 Model processing

We divided the dataset into a 4:1 ratio, with 2400 data points used as the training set and 600 data points used as the prediction set. To ensure that both subsets faithfully reflect the balanced class distribution resulting from our data augmentation process, we performed stratified random sampling on the full dataset using the class label as the stratification key. This study employs machine learning algorithms such as Random Forest (27), SVM (28), XGBoost (29), and MLP (30), comparing their prediction results with the proposed model.

#### 2.3.1 Basic learning models

Random Forest: Random Forest (27) is an ensemble learning method that improves the accuracy and stability of a model by constructing multiple decision trees and combining their predictions. The training set for each tree is obtained through Bootstrap Sampling, randomly sampling from the original training set. Each tree is trained in a different subset, and each node is split using a fraction of the features to minimize overfitting.

Formula:

Information Gain (for selecting the best split):


$$I\left(G,D\right)=H\left(D\right)-\sum_{k=1}^{K}\frac{\left|D_{k}\right|}{\left|D\right|}H\left(D_{k}\right)$$

where *H*(*D*) is the entropy of the dataset (*D*), and *D_k_* is the subset after partitioning by feature (*k*).

Entropy:


$$H\left(D\right)=-\sum_{i=1}^{n}p_{i}\;\text{log }p_{i}$$

where *p_i_* is the probability of class

Out-of-bag error:


$$\text{OOB Error}=\frac{1}{n}\sum_{i=1}^{n}I\left(y_{i}\neq {\hat{y}}_{i}^{\text{OOB}}\right)$$

When using the Random Forest classifier in **Algorithm 1**, the process begins with Bootstrap Sampling to create multiple subsets from the original training set. Each decision tree in the forest is trained on a different subset, enhancing the model’s robustness and reducing the risk of overfitting. We select the optimal features and split points for each node within these trees to maximize information gain. Information gain is calculated based on the reduction in entropy, ensuring that each split improves the purity of the subsets. This process of selecting features and splitting points is crucial, as it determines the effectiveness of the Random Forest in distinguishing between different classes. The Random Forest algorithm also calculates out-of-bag (OOB) error, which provides an unbiased estimate of the model performance and helps assess its accuracy.

Algorithm 1 **Machine learning classifier processing process.**

**Input:** Original datasets
**Output:** accuracy, precision, recall, F1 and AUC
**1foreach** *Classifier in* {*Random Forest, XGBoost, SVM*} **do**

**2** Initialize Classifier Object;
**3 foreach** *Unique class label in the target variable* **do**

**4**  Convert the target variable to the binary format;
**5**  Split the original datasets into training and   testing sets;
**6**  Initialize the normalizer and perform data  standardization;
**7**  Use training data to train each classifier;
**8 end**

**9 if** *The Classifier is Random Forest* **then**

**10 foreach** *Decision tree in the Random Forest* **do**

**11**  Randomly select samples from the training set;
**12  end**

**13   foreach** *Node in the tree* **do**

**14**   Select optimal features and splitting points to     maximize information gain;
**15  end**

**16  end**

**17 if** *The Classifier is XGBoost* **then**

**18  foreach** *Decision tree in XGBoost* **do**

**19**   Calculate the gradient and Hessian for each node;
**20**   Select the optimal features and splitting points to    minimize the loss function;
**21  end**

**22  end**

**23 if** *The Classifier is SVM* **then**

**24**   Solve optimization problems to find the hyperplane    with the maximum margin;
**25**   *w* ×*x*+*b* = 0;
**26 end**

**27** Use the trained classifier to predict test data;
**28 end**

**29 return** accuracy, precision, recall, F1 score, and AUC

**XGBoost:** XGBoost (29) is an optimized Gradient Boosting Decision Tree (GBDT) algorithm known for its speed and performance. The loss function is minimized by constructing the tree step-by-step and adding a new tree at each step to correct the error from the previous step. A regularization term is included to prevent overfitting.

Formula: Loss function:


$$L\left(\theta \right)=\sum_{i=1}^{n}l\left(y_{i},{\hat{y}}_{i}\right)+\sum_{k=1}^{K}\text{Ω}\left(f_{k}\right)$$

where $\text{Ω}\left(f_{k}\right)$ is $\gamma T+\frac{1}{2}\lambda \sum_{j=1}^{T}w_{j}^{2}$ , γ and λ are the regularization parameters.

Incremental lift:


$${\hat{y}}_{i}^{\left(t\right)}={\hat{y}}_{i}^{\left(t-1\right)}+\eta f_{t}\left(x_{i}\right)$$

where ${\hat{y}}_{i}^{\left(t\right)}$ is the predicted value in round (*t*), *η* is the learning rate, and *f_t_* is the weak learner at round *t*.

Split node selection:


$$\text{Gain}=\frac{1}{2}\left[\frac{G_{L}^{2}}{H_{L}+\lambda }+\frac{G_{R}^{2}}{H_{R}+\lambda }-\frac{{\left(G_{L}+G_{R}\right)}^{2}}{H_{L}+H_{R}+\lambda }\right]$$

where *G_L_* and *G_R_* are the gradient sums of the left and right child nodes, respectively, and *H_L_* and *H_R_* are the second-order gradient sums of the left and right child nodes, respectively.

In the case of the XGBoost classifier in **Algorithm 1**, the approach is slightly different with Random Forest. XGBoost, an optimized implementation of Gradient Boosting, builds trees sequentially, where each new tree aims to correct the errors of the previous ones. For each decision tree, we calculate the gradient and Hessian of each node. The gradient represents the first derivative of the loss function concerning the predicted value, indicating the direction in which the prediction should move to reduce error. The Hessian, the second derivative, provides information about the curvature of the loss function, helping to adjust the step size for the updates. XGBoost effectively enhances the model’s accuracy while including a regularization term to prevent overfitting by selecting the optimal features and split points to minimize the loss function. Using gradient and Hessian makes XGBoost particularly efficient and powerful in handling large-scale and complex datasets. Additionally, the algorithm evaluates the gain of each split, ensuring that each new tree contributes effectively to reducing the overall prediction error.

**SVM:** Support Vector Machines (28) maximize the classification boundaries by finding the best hyperplane to separate the data.

Find a hyperplane in high-dimensional space such that the sample points of different classes are as far away from that hyperplane as possible. Non-linearly differentiable data can be handled by kernel tricks.

Formula:

Hard spacing is maximized:


$$\underset{\text{w},b}{\min}\frac{1}{2}\|\text{w}{\|}^{2}\text{ subject to }y_{i}\left(\text{w}\cdot {\text{x}}_{i}+b\right)\geq 1$$

where **w** is the weight vector, *b* is the bias, and *y_i_* is the category label.

Soft interval maximization (allowing misclassification):


$$\underset{\text{w},b,\xi }{\min}\frac{1}{2}\|\text{w}{\|}^{2}+C\sum_{i=1}^{n}\xi_{i}\text{ subject to }y_{i}\left(\text{w}\cdot {\text{x}}_{i}+b\right)\geq \xi 1{-}_{i}$$

where *ξ_i_* is the slack variable, *C* is the penalty parameter.

Kernel function (maps data to a higher-dimensional space):


$$K\left({\text{x}}_{i},{\text{x}}_{j}\right)=\phi \left({\text{x}}_{i}\right)\cdot \phi \left({\text{x}}_{j}\right)$$

Commonly used kernel functions include linear kernel, polynomial kernel, radial basis kernel (RBF), etc.

For the SVM classifier in **Algorithm 1**, the focus is finding the hyperplane that best separates the data into different classes. This is achieved by solving an optimization problem that maximizes the margin between the closest points of the different classes, known as support vectors. The larger the margin, the better the generalization capability of the SVM. In cases where the data are not linearly separable, SVM employs kernel tricks to map the data into a higher-dimensional space, where a linear hyperplane can perform the separation. Commonly used kernels include the linear kernel, polynomial kernel, and RBF kernel, each transforming the data in specific ways to reveal patterns that are not visible in the original feature space. The choice of kernel and the parameters used in the SVM model significantly influence its performance and ability to generalize well to unseen data.

MLP: Multilayer Perceptron (30) learn complex patterns and features through multiple hidden layers and a large number of neurons. They consist of several layers (input, hidden, and output), each containing several neurons.

Formula:

Forward propagation:


$$a^{\left(l\right)}=f\left(\sum_{j=1}^{n_{l-1}}w_{ij}^{\left(l\right)}a_{j}^{\left(l-1\right)}+b_{i}^{\left(l\right)}\right)$$

where *a* ^(^ *^l^* ^)^ is the activation value of the *l*-th layer. *w* and *b* are the weight and bias respectively.

Activation function: Commonly used activation functions include Sigmoid, ReLU (Rectified Linear Unit), Tanh, and others.

Loss function (cross-entropy):


$$L=-\sum_{i=1}^{n}\left[y_{i}\text{log }(\hat{y_{i}})+\left(1-y_{i}\right)\text{log }(1-\hat{y_{i}})\right]$$

Reverse propagation:


$$\frac{\partial L}{\partial w_{ij}^{\left(l\right)}}=\delta_{i}^{\left(l\right)}a_{j}^{\left(l-1\right)}$$

where $\delta_{i}^{\left(l\right)}$ is the error term in layer *l*.

**Algorithm 2** corresponds to the MLP classifier. For each hidden layer, we use the function *z* = *wx*+*b* and *a* = *activation function*(*z*). For the output layer, we use the function: *z* = *wx*+*b* and *y* = *softmax*(*z*). For all algorithms, we choose the industry-recognized accuracy, precision, recall, F1 score, and AUC to evaluate the accuracy of the prediction results.

Algorithm 2 **Multilayer perceptron processing process.**

**Input:** Original datasets
**Output:** accuracy, precision, recall, F1 and AUC
**1** Initialize MLP Classifier Object;
**2 foreach** *unique class label in the target variable* **do**

**3** Convert the target variable to the binary format of the current class label;
**4** Split the original datasets into training and  testing sets;
**5** Initialize the normalizer and perform data  standardization;
**6** Use training data to trainan MLP classifier;
**7 foreach** *hidden layer in the network* **do**

**8**  Compute *z* = *w* × *x* + *b*;
**9**  Apply activation function: *a* = *activation*_*function*   (*z*);
**10 end**

**11 for** *the output layer* **do**

**12**  Compute *z* = *w* × *x* + *b*;
**13**  Compute predictions: *y* = *softmax*(*z*);
**14 end**

**15** Use the trained classifier to predict test data;
**16** Calculate the accuracy, precision, recall, F1score,   and AUC of prediction results;
**17** Obtain feature importance;
**18** Store the result of the current class label in a   DataFrame;
**19 end**

**20 return** accuracy, precision, recall, F1score, and AUC

#### 2.3.2 Stacking classifier with Bayesian optimization

Stacking classifiers: Stacking classifiers (31) are ensemble learning methods combining multiple base classifiers using a meta-classifier to optimize final predictions. They leverage the strengths of diverse models to enhance performance and generalization. A stacking classifier consists of base classifiers and a meta-classifier.

Formula:

Base Classifier Prediction:


$${\hat{y}}_{i}^{\left(j\right)}=f_{j}\left(X\right),\;j=1,2,\ldots ,k$$

where ${\hat{y}}_{i}^{\left(j\right)}$ is the prediction of the *j*-th base classifier for input *X*, and *k* is the total number of base classifiers.

Meta-Classifier Input:


$$Z=\left[\begin{array}{cccc} {\hat{y}}_{1}^{\left(1\right)} & {\hat{y}}_{2}^{\left(1\right)} & \ldots & {\hat{y}}_{n}^{\left(1\right)}\\ {\hat{y}}_{1}^{\left(2\right)} & {\hat{y}}_{2}^{\left(2\right)} & \ldots & {\hat{y}}_{n}^{\left(2\right)}\\ \vdots & \vdots & \ddots & \vdots \\ {\hat{y}}_{1}^{\left(k\right)} & {\hat{y}}_{2}^{\left(k\right)} & \ldots & {\hat{y}}_{n}^{\left(k\right)} \end{array}\right]$$

where **Z** represents the output matrix from all base classifiers, serving as input to the meta-classifier.

Meta-Classifier Training:


$${\hat{y}}_{final}=g\left(\text{Z},\theta \right)$$

where *g* represents the meta-classifier, and *θ* refers to its parameters.

**Implementation details.** **Algorithm 3** corresponds to the stacking classifier. Based on the experimental analysis of basic learning models in Section 3.2, we selected three basic models that perform well on specific classes to form the stacking classifier. The stacking process combines base classifiers’ strengths while addressing their weaknesses. For each base classifier, predictions are generated and combined as input features for the meta-classifier. In the experimental section, we will compare several meta-classifiers and determine which is the best one in our dataset.

Algorithm 3 **Stacking Classifier Processing Process.**

**Input:** Original datasets
**Output:** accuracy, precision, recall, F1 and AUC
1 Initialize base classifiers: Random Forest (RF), XGBoost (XGB), and Support Vector Machine (SVM);
2 Initialize meta-classifier;
3 **foreach** *unique class label in the target variable* **do**

4 Convert the target variable to the binary format of  the current class label;
5 Split the original datasets into training and  testing sets;
6 Initialize the normalizer and perform data  standardization;
7 **foreach** *base classifier in [RF, XGB, SVM]* **do**

8  Use training data to train the base classifier;
9  Obtain predictions from the base classifier for the   training and testing sets;
10 Store the predictions as features for the   meta-classifier;
11 end
12 Use the meta-classifier to train on the base  classifiers’ predictions;
13 Use the trained stacking model to predict the test  data;
14 Calculate the accuracy, precision, recall, F1   score, and AUC of prediction results;
15 end
16 **return** accuracy, precision, recall, F1 score, and AUC

**•Base classifiers:** Random Forest, XGBoost, and SVM. Each classifier is trained independently on the training dataset.

**•Meta-classifier:** Random Forest, XGBoost, SVM, Logistic Regression, and Stacking Classifier. It learns from the outputs of the base classifiers to make the final prediction.

For evaluation, we use industry-recognized metrics such as accuracy, precision, recall, F1 score, and AUC to measure the performance of the stacking classifier.

**Bayesian optimization:** Bayesian Optimization is a sequential global optimization framework that is particularly effective for tuning hyperparameters in complex machine learning models, such as stacking classifiers. A probabilistic surrogate model approximates the expensive black-box objective function in this framework. An acquisition function then guides the search for the optimum by balancing exploring uncertain regions and exploiting regions known to yield favorable outcomes. This methodology is highly efficient in scenarios where each function evaluation (such as training a stacking classifier with various hyperparameter configurations) is computationally expensive.

**Implementation details.** Firstly, we define the search space for the base classifiers (Random Forest, XGBoost, SVM) in **Table 3**. For Random Forest, choosing 50–200 trees manages the trade-off between high variance from too few estimators and the diminishing returns on variance reduction with excessive trees, while a maximum depth of 3–20 prevents both underfitting and overfitting by regulating model complexity (32). Similarly, setting the minimum samples required for splitting between 2 and 10 ensures robust partitioning without leading to overly granular splits (33). In XGBoost, the interval of 50–200 boosting rounds typically allows sufficient convergence without incurring excessive computational cost or overfittin (29), and sampling the learning rate on a log-uniform scale between 0.01 and 0.30 efficiently explores the spectrum between rapid but potentially unstable convergence and slower, more stable learning (34). The maximum tree depth range in XGBoost, also set between 3 and 20 and provides a comparable mechanism for controlling model complexity (29). For the Support Vector Machine, the regularization parameter C is explored on a log-uniform scale from 0.001 to 1 to balance the trade-off between margin maximization and classification error (35), while restricting the kernel function to either linear or RBF ensures applicability to both linearly separable data and datasets exhibiting nonlinear patterns (36).

**Table 3:** Hyperparameter of Bayesian optimization.

Classifier	Hyperparameter	Type	Range/Categories
Random Forest	ensemble size	Integer	50 to 200
	tree depth	Integer	3 to 20
	node splitting	Integer	2 to 10
XGBoost	number of boosting rounds	Integer	50 to 200
	learning rate	Log-uniform	0.01 to 0.3
	tree complexity	Integer	3 to 20
SVM	penalty term	Log-uniform	0.001 to 1
	kernel function	Categorical	{linear, RBF}

Integer: Discrete values within a specified range.

Log-uniform: Continuous values sampled logarithmically within the range.

Categorical: Discrete options from a predefined set.

Secondly, assuming the defined hyperparameter search space is denoted as Θ, we initialize a Bayesian optimization process over this space. We suppose *H_t_* as the set of observations up to the *t*-th trial including the hyperparameter configuration *θ_t_* corresponding to the highest 5-fold cross-validated accuracy *A_t_*.


$$A_{t}=Accuracy\left(StackingClassifier\left(\theta_{t}\right)\right)$$

At step *t*, we employ the Tree-structured Parzen Estimator (TPE) as the Surrogate Model to generate the next set of hyperparameters. The function of this process can be represented as


$$\theta_{t+1}=\underset{\theta \in \text{Θ}}{\text{arg max α}}(\theta |H_{t})$$

The acquisition function *α* is used to determine the next hyperparameter point to evaluate. Our acquisition function is based on Expected Improvement, which is equivalent to the ratio of the distribution of good-performing hyperparameters *g*(*θ*) to that of poor-performing ones *l*(*θ*). When this ratio $\frac{g\left(\theta \right)}{l\left(\theta \right)}$ is large, it indicates that the sampling point lies in a region of good performance, thus holding greater potential for improvement. Given that our parameters include both continuous and discrete types, for continuous variables, we utilize Gaussian kernel density estimation, while for discrete hyperparameters, we apply histogram estimation. After obtaining the *θ_t_*_+1_, we compute the *A_t_*_+1_ and update the optimal set of observations if *A_t_*_+1_ is better than the previous best value. After *T* trials, the best-performing hyperparameters are selected, improving the accuracy and robustness. *T* are set to 5000 in our experiment.

## 3 Results and discussions

In this section, we first trained the base learning models on the imbalanced dataset to demonstrate the significant impact of data imbalance on model performance. Subsequently, we conducted experiments using the DCD dataset based on data augmentation and compared the performance of base learning models and different stacking classifiers. Based on the performance analysis of different models, we selected the best model for Bayesian optimization to further enhance its performance. Finally, we performed feature importance analysis on the model, and by filtering out unimportant features, we were able to further improve the model’s classification performance and reduced the cost of medical treatment.

### 3.1 The impact of class imbalance

**Figure 1:**
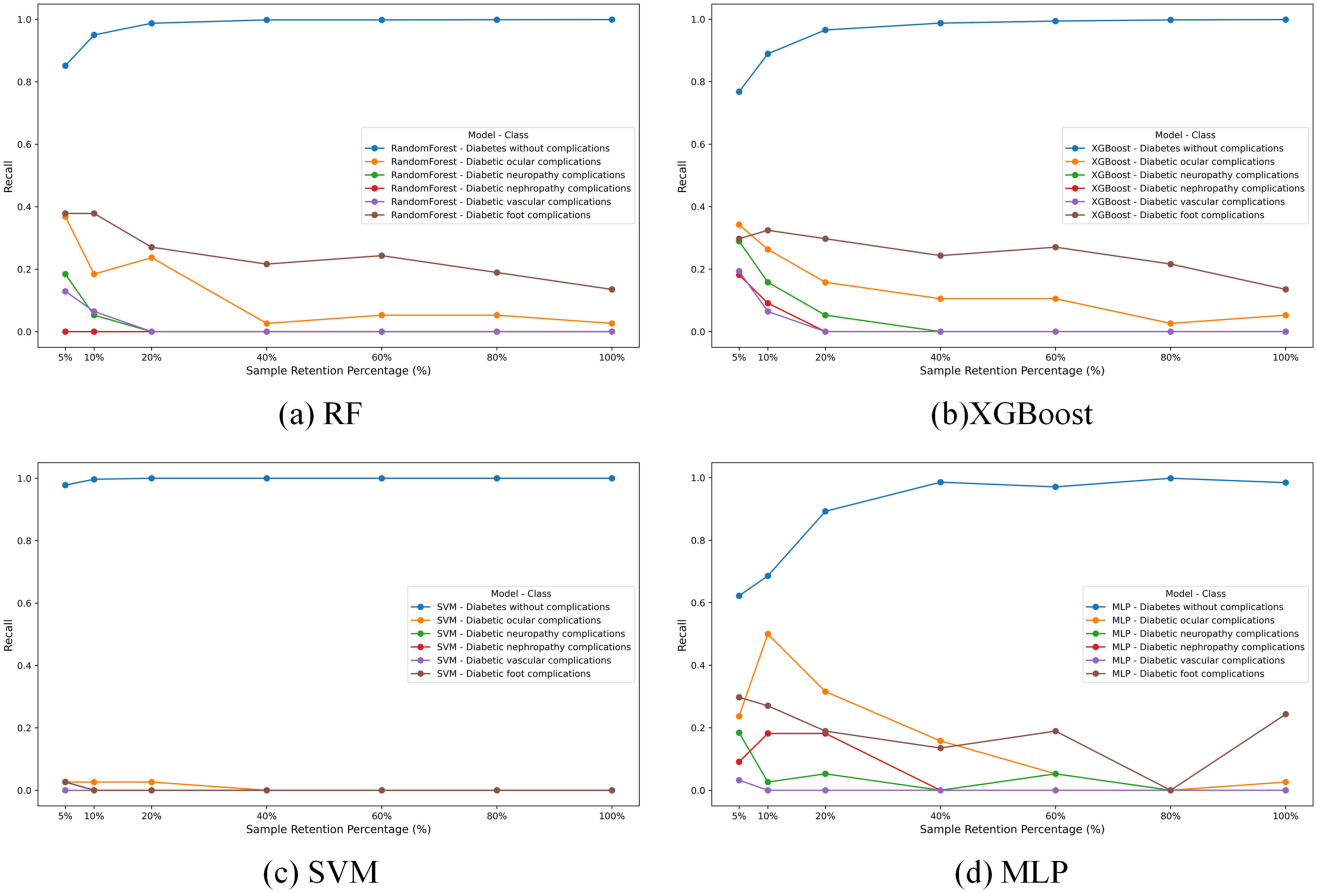
The Recall of different models **(a–d)** on the imbalanced dataset.

In our study, we trained four different machine learning models-RF, XGBoost, SVM, and MLP—on a raw imbalanced dataset. The recall results for these models across different classes are illustrated in **Figure 1**, which reveals a pronounced performance disparity attributable to the imbalance of the dataset. The first class, due to its prominent data volume, achieves an accuracy close to 1, while the recall rates of other models are generally below 0.4. Particularly, SVM is the most affected, with recall values close to 0.

To investigate the impact of this imbalance, we conducted a sensitive analysis by varying the sample retention percentage of the “Diabetes without complications” category (Class 0), as depicted in **Figure 1**. We selected seven different proportions to retain the data of the Class 0. Overall, for most models, the performance on other classes tends to decrease as the data volume of the Class 0 increases.

In summary, data imbalance leads to the near-total failure of basic learning models in predicting other classes. Therefore, performing data augmentation on other classes and controlling the data volume of the Class 0 is crucial for balancing the overall performance of the model.

### 3.2 The results on DCD dataset

#### 3.2.1 The results of basic learning models

**Figure 2:**
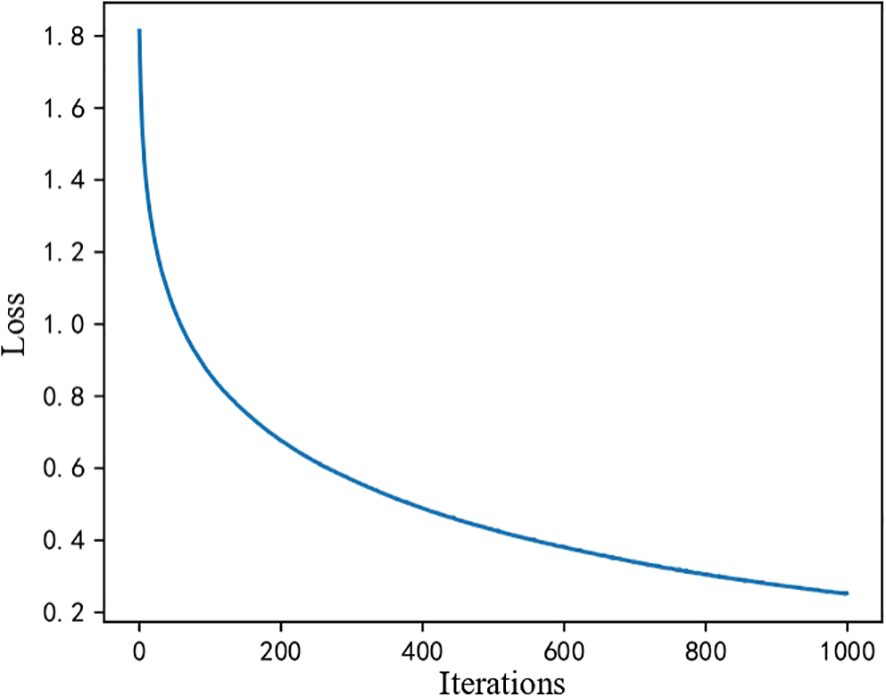
MLP convergence curve.

First, because machine learning models do not involve iteration, there is no loss function change curve. However, for the MLP processing flow described in Section 2, we obtained the loss function curve of the iteration during training, as shown in **Figure 2**. The loss value is high in the initial stage (the first 200 iterations). Still, it drops rapidly from nearly 1.8 to about 0.2, indicating that the model has learned a significant amount of helpful information early in the training process. In the middle stage, the loss value continues to decrease. However, the rate of decrease slows, suggesting that while the model is still learning, the performance improvements brought by each iteration are diminishing. In the final convergence stage, the curve stabilizes, and the loss value remains unchanged, indicating that the model has reached a steady state where further training yields minimal improvement. This overall trend demonstrates that the model converges effectively without evident overfitting or underfitting.

The prediction results of the basic learning models are shown in **Table 4**. The prediction accuracy of the various models for each type of complication is high, all exceeding 80%. The precision in predicting the category of no complications in diabetes is the lowest among the categories, with the highest accuracy achieved using the Random Forest at 86.83%. For the other complication categories, except for the SVM in predicting vascular complications of diabetes and the MLP in predicting neurological complications of diabetes, the prediction accuracy of all other classifiers exceeds 90%. We bold the highest values of several indicators with the highest prediction accuracy in each category. It is not difficult to find that XGBoost demonstrates the best overall performance among the base learning models.

**Table 4:** Prediction results of basic learners.

Complication types	Classifiers	Accuracy	Recall	F1	AUC
No complications in diabetes	Random Forest	**86.83%**	28.71%	42.34%	**90.04%**
Ocular complications of diabetes		93.83%	64.95%	77.30%	94.74%
Neurological complications of diabetes		90.00%	43.69%	60.00%	93.60%
Complications of diabetes nephropathy		97.00%	82.98%	89.66%	**99.59%**
Vascular complications of diabetes		**93.00%**	55.91%	71.23%	**97.08%**
Foot complications of diabetes		**94.83%**	72.32%	83.94%	95.91%
No complications in diabetes	XGBoost	86.67%	**43.56%**	**52.38%**	85.71%
Ocular complications of diabetes		**95.33%**	**75.26%**	**83.91%**	**96.27%**
Neurological complications of diabetes		**91.83%**	**60.19%**	**71.68%**	**94.39%**
Complications of diabetes nephropathy		**98.33%**	**91.49%**	**94.51%**	99.54%
Vascular complications of diabetes		92.83%	**67.74%**	**74.56%**	94.91%
Foot complications of diabetes		**94.83%**	**75.00%**	**84.42%**	**96.11%**
No complications in diabetes	SVM	83.67%	3.96%	7.55%	78.57%
Ocular complications of diabetes		89.33%	44.33%	57.33%	89.45%
Neurological complications of diabetes		83.83%	5.83%	11.01%	82.78%
Complications of diabetes nephropathy		92.00%	55.32%	68.42%	95.04%
Vascular complications of diabetes		86.00%	10.75%	19.23%	88.93%
Foot complications of diabetes		91.00%	54.46%	69.32%	89.23%
No complications in diabetes	MLP	85.17%	34.65%	44.03%	84.64%
Ocular complications of diabetes		91.67%	60.82%	70.24%	92.16%
Neurological complications of diabetes		87.17%	39.81%	51.57%	90.88%
Complications of diabetes nephropathy		96.50%	90.43%	89.01%	99.18%
Vascular complications of diabetes		90.17%	62.37%	66.29%	92.70%
Foot complications of diabetes		91.33%	60.71%	72.34%	91.42%

Bold means the optimal values among different methods for the same evaluation metric.

**Figure 3:**
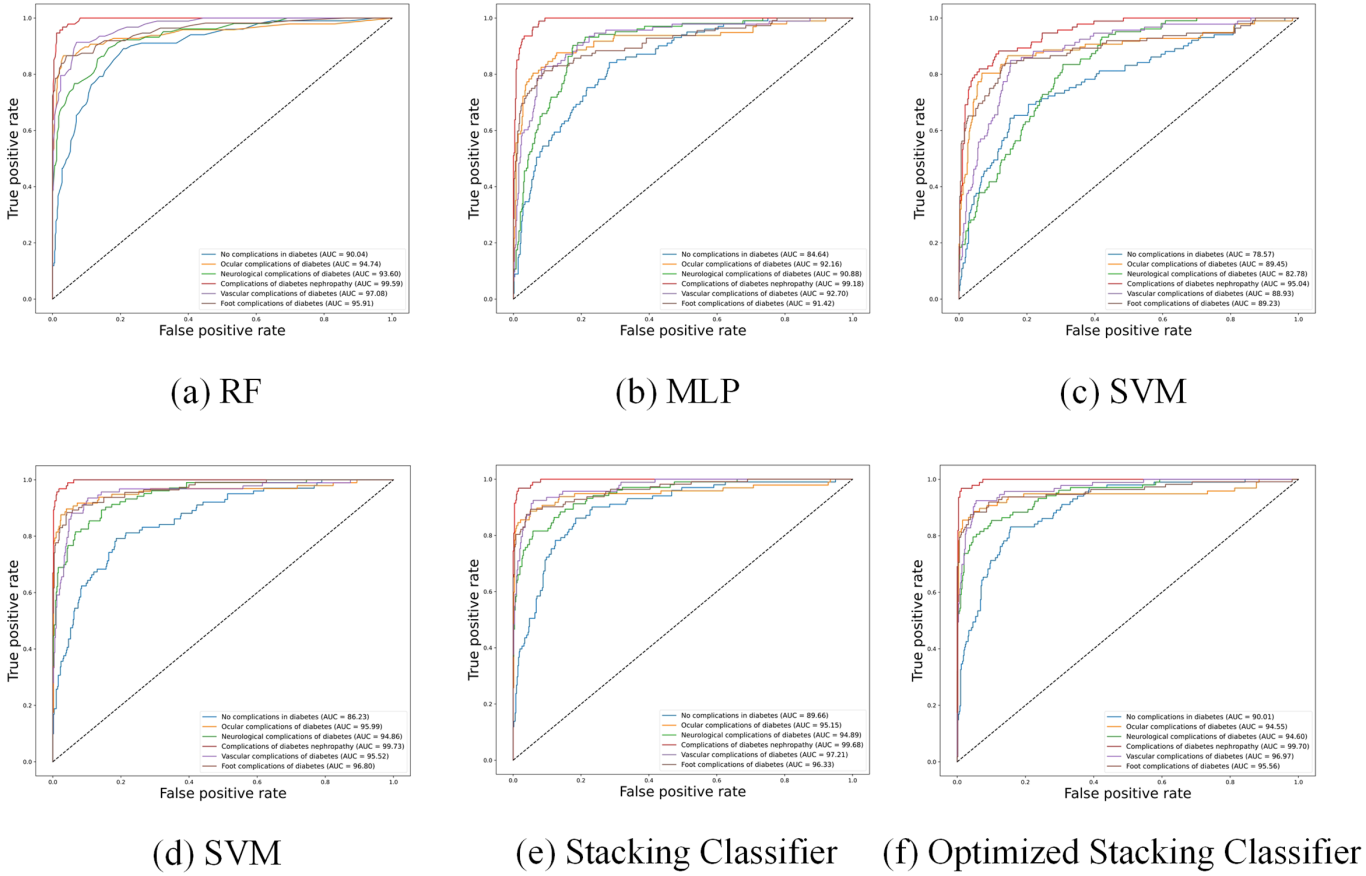
ROC curves of different classifiers **(a–f)**.

Furthermore, **Figure 3** shows the Receiver Operating Characteristic (ROC) curves of different baseline classifiers. From the figure, it is apparent that the accuracy of the models in predicting diabetic complications is high, with large AUC. The ROC curves indicate that Random Forest, XGBoost, and MLP outperform SVM, especially in predicting complications of diabetes nephropathy. However, for other complications, MLP’s performance is noticeably inferior to that of Random Forest and XGBoost.

#### 3.2.2 The results of stacking classifier

Based on the performance analysis of the base learning models, we found that Random Forest, XGBoost, and SVM all demonstrated good predictive performance in specific classes, while MLP exhibited relatively poorer performance. Assuming the meta-classifier is the logistic regression, we compared the basic classifiers with and without MLP in the **Table 5**. It was found that the performance of the basic classifiers with MLP decreased.

**Table 5:** Prediction results of stacking learning with and without MLP.

Complication Types	Classifiers	Accuracy	Recall	F1	AUC
No complications in diabetes	Stacking Classifier with MLP (logits)	87.17%	49.51%	56.50%	**89.96%**
Ocular complications of diabetes		96.17%	80.41%	87.15%	95.14%
Neurological complications of diabetes		92.27%	67.90%	76.44%	94.36%
Complications of diabetes nephropathy		98.00%	**92.55%**	93.55%	99.33%
Vascular complications of diabetes		94.20%	81.72%	83.02%	97.06%
Foot complications of diabetes		**95.16%**	**77.68%**	**85.71%**	96.08%
No complications in diabetes	Stacking Classifier without MLP (logits)	**87.67%**	**50.50%**	**57.95%**	89.66%
Ocular complications of diabetes		**96.50%**	**81.44%**	**88.27%**	**95.15%**
Neurological complications of diabetes		**92.83%**	**67.96%**	**76.50%**	**94.89%**
Complications of diabetes nephropathy		**98.17%**	**92.55%**	**94.05%**	**99.68%**
Vascular complications of diabetes		**94.83%**	**82.80%**	**83.24%**	**97.21%**
Foot complications of diabetes		**95.16%**	**77.68%**	**85.71%**	**96.33%**

Bold means the optimal values among different methods for the same evaluation metric.

Therefore, we selected Random Forest, XGBoost, and SVM as the base classifiers for the stacking classifier and conducted further research on different meta-classifiers. As shown in **Table 6**, we evaluated six different meta-classifiers and presented their predictive performance on each class. When SVM was used as the meta-classifier, its performance was poor, especially for the class “Vascular complications of diabetes,” which remained below 90%. The averaging method showed significantly better accuracy than Random Forest. XGBoost achieved the best results on the classes “Complications of diabetes nephropathy” and “Foot complications of diabetes,” with accuracies of 98.33% and 95.16%, respectively. When logistic regression was used as the meta-classifier, the overall performance improved markedly. Although the AUC values were not the highest, they were very close to the highest values. To further explore the potential for improvement, we conducted additional experiments using the best stacking classifier with logistic regression as the meta-classifier. We found that while there were improvements in “Complications of diabetes nephropathy” and “Foot complications of diabetes,” the performance on most other classes declined. Therefore, we ultimately chose logistic regression as the meta-classifier.

**Table 6:** Prediction results of stacking learning.

Complication types	Classifiers	Accuracy	Recall	F1	AUC
No complications in diabetes	Stacking Classifier (Averaging)	87.00%	37.62%	49.35%	88.82%
Ocular complications of diabetes		95.67%	75.26%	84.88%	94.88%
Neurological complications of diabetes		90.00%	45.63%	61.04%	95.10%
Complications of diabetes nephropathy		97.67%	88.30%	92.22%	99.37%
Vascular complications of diabetes		93.17%	61.29%	73.55%	96.72%
Foot complications of diabetes		94.33%	72.32%	82.65%	95.84%
No complications in diabetes	Stacking Classifier (RF)	86.83%	28.71%	42.34%	**90.04%**
Ocular complications of diabetes		93.83%	64.95%	77.30%	94.74%
Neurological complications of diabetes		90.00%	43.69%	60.00%	93.60%
Complications of diabetes nephropathy		97.00%	82.98%	89.66%	99.59%
Vascular complications of diabetes		93.00%	55.91%	71.23%	97.08%
Foot complications of diabetes		94.83%	72.32%	83.94%	95.91%
No complications in diabetes	Stacking Classifier (XGBoost)	86.33%	39.60%	49.38%	86.23%
Ocular complications of diabetes		96.00%	79.38%	86.52%	**95.99%**
Neurological complications of diabetes		92.50%	63.11%	74.29%	94.86%
Complications of diabetes nephropathy		**98.33%**	92.55%	94.57%	**99.73%**
Vascular complications of diabetes		92.16%	66.67%	72.51%	95.52%
Foot complications of diabetes		**95.16%**	76.79%	85.57%	**96.80%**
No complications in diabetes	Stacking Classifier (SVM)	83.67%	3.96%	7.54%	78.57%
Ocular complications of diabetes		83.83%	5.82%	11.01%	82.76%
Neurological complications of diabetes		83.83%	5.83%	11.01%	**82.78%**
Complications of diabetes nephropathy		92.00%	55.32%	68.42%	95.03%
Vascular complications of diabetes		86.00%	10.75%	19.23%	88.93%
Foot complications of diabetes		91.00%	54.46%	69.32%	89.23%
No complications in diabetes	Stacking Classifier (logits)	**87.67%**	**50.50%**	**57.95%**	89.66%
Ocular complications of diabetes		**96.50%**	**81.44%**	**88.27%**	95.15%
Neurological complications of diabetes		**92.83%**	**67.96%**	76.50%	94.89%
Complications of diabetes nephropathy		98.17%	92.55%	94.05%	99.68%
Vascular complications of diabetes		**94.83%**	**82.80%**	**83.24%**	**97.21%**
Foot complications of diabetes		**95.16%**	**77.68%**	85.71%	96.33%
No complications in diabetes	Stacking Classifier (Stacking)	86.50%	44.55%	52.63%	85.47%
Ocular complications of diabetes		95.50%	76.29%	84.57%	95.33%
Neurological complications of diabetes		92.67%	66.02%	**77.56%**	93.95%
Complications of diabetes nephropathy		**98.33%**	**93.62%**	**94.62%**	98.00%
Vascular complications of diabetes		94.50%	81.72%	82.16%	94.65%
Foot complications of diabetes		**95.33%**	**77.68%**	**86.14%**	93.35%

Bold means the optimal values among different methods for the same evaluation metric.

**Figure 3** shows the ROC curve of the Stacking Classifier with logistic regression, which combines the strengths of multiple baseline classifiers to achieve superior performance. The figure shows that the stacking classifier with logistic regression consistently demonstrates a higher AUC across all diabetic complication categories, indicating its robustness and better generalization capability than individual classifiers.

#### 3.2.3 The results of stacking classifier with Bayesian optimization

In this subsection, we optimized the stacking classifier with the best performance obtained previously using the Bayesian optimization algorithm, and the results are presented in **Table 7**. The stacking classifier and optimized version provide the highest values for all other metrics. In particular, when predicting.

**Table 7:** Prediction results of stacking classifier with Bayesian optimization.

Complication types	Classifiers	Accuracy	Recall	F1	AUC
No complications in diabetes	Stacking Classifier (logits) + Bayesian Optimization	**88.33%**	**50.50%**	**59.30%**	**90.20%**
Ocular complications of diabetes		**96.50%**	**81.44%**	**88.27%**	95.15%
Neurological complications of diabetes		**93.50%**	**71.84%**	**79.14%**	**95.21%**
Complications of diabetes nephropathy		**98.33%**	**93.62%**	**94.62%**	**99.71%**
Vascular complications of diabetes		**94.83%**	**82.80%**	**83.79%**	**97.21%**
Foot complications of diabetes		**95.67%**	**79.46%**	**87.25%**	95.84%

Bold means the optimal values among different methods for the same evaluation metric.

“neurological complications of diabetes”, precision reaches 93.50%, with an AUC of 95.21%, and both F1 and recall values exceed 70%, significantly outperforming all other methods. Although the AUC values in predicting “Ocular complications of diabetes” and “Foot complications of diabetes” are lower than optimal values, the differences are only 0.17% and 0.96% respectively. However, the recall and F1-score values of optimized stacking classifier are highest. **Figure 3** further illustrates that the stacking classifier is more robust after training by Bayesian optimization. Therefore, the stacking classifier with Bayesian optimization is identified as the optimal model to predict diabetic complications, and we will use this method as the basis for subsequent optimization.

**Figure 4:**
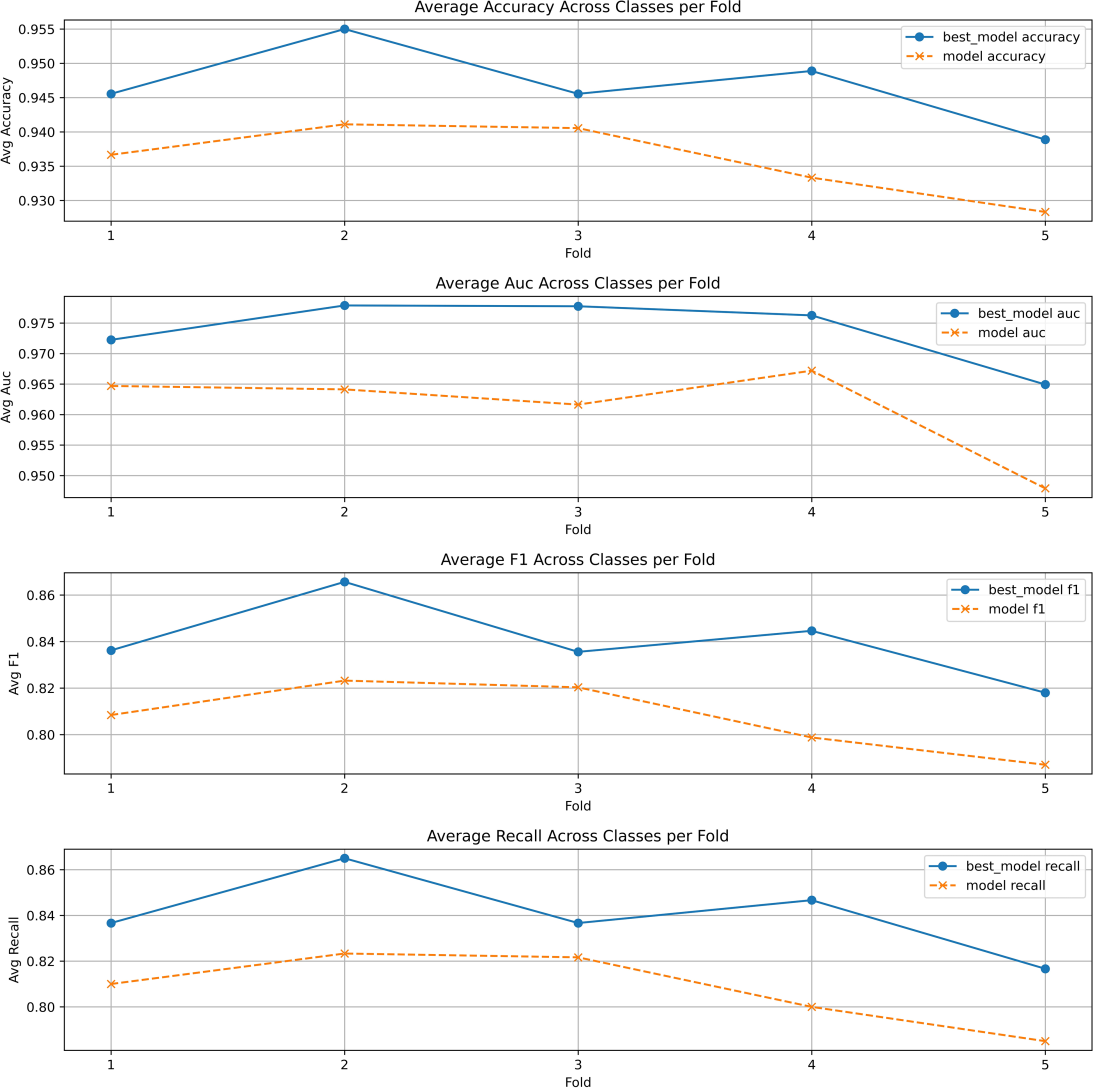
The 5-fold cross validation of stacking classifier with and without Bayesian optimization.

To avoid the Bayesian optimization favoring a particular train/test split and to further demonstrate the effectiveness of the model based on Bayesian optimization, we introduced 5-fold cross-validation. The dataset was divided into five different train and test splits, and the optimal hyperparameter combinations were applied to each split separately. Ultimately, the average metrics for each fold were calculated and compared with the unoptimized model in the **Figure 4**. It can be observed that the optimized stacking classifier showed varying degrees of improvement in average performance in each split, which proves that the optimal parameter configuration found by Bayesian optimization has generalizability.

#### 3.2.4 The analysis of feature importance

Our work also considers the relevance of features. Since selected features are the indicators most frequently tested in the hospital, our objective is to reduce the cost of patient testing by removing irrelevant features. This approach allows for more accurate predictions of diabetic complications using fewer indicators.

**Figure 5:**
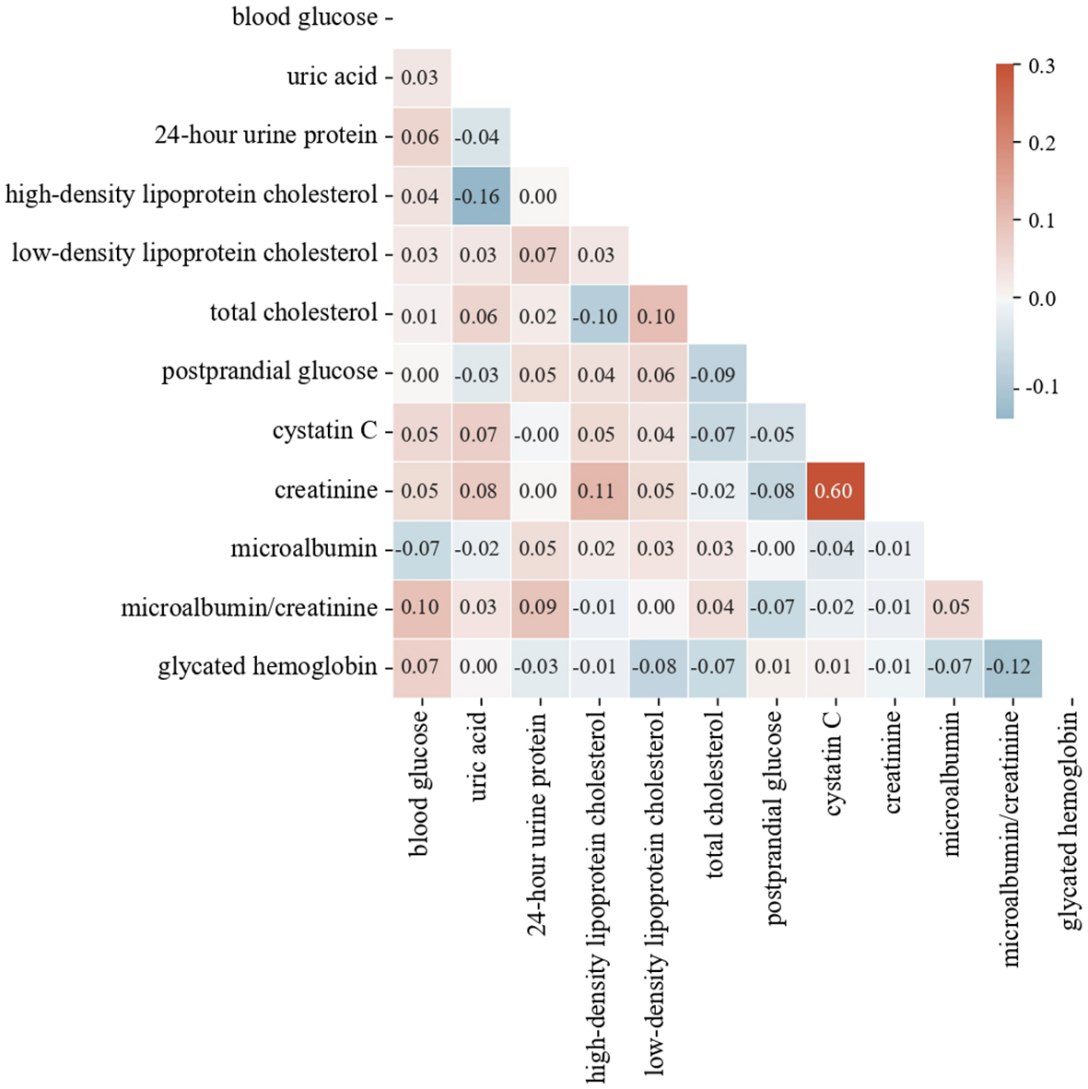
The heat map of each feature.

This study uses the Pearson correlation coefficient matrix to analyze the correlation between diabetes features and removes the highly correlated features based on the results. The Pearson correlation coefficient (37) measures the degree of linear correlation between two variables, calculated as follows:


$$r=\frac{\sum_{i=1}^{n}(x_{i}-\bar{x})\left(y_{i}-\bar{y}\right)}{\sqrt{\sum_{i=1}^{n}{\left(x_{i}-\bar{x}\right)}^{2}\sum_{i=1}^{n}{\left(y_{i}-\bar{y}\right)}^{2}}}$$

where *r* is the Pearson correlation coefficient, *x_i_* and *y_i_* are the *i*-th observations of the two variables, and $\bar{x}$ and $\bar{y}$ are the means of the two variables. The Pearson correlation coefficient ranges from -1 to 1. Values closer to 1 or -1 indicate stronger linear correlations, while values closer to 0 indicate weaker correlations. Generally, when the absolute value of the correlation coefficient exceeds 0.85, the two features are considered to have a very high correlation. The heat map in **Figure 5** shows the highest correlation is 0.6, indicating no need to delete any features. Based on feature importance, we remove less important features to observe changes in model performance.

We list the feature importance of a single model and the average importance of multiple models in **Table 8**. We found that the feature importance’s ranks of XGBoost and RF are similiar to the average version, and the “postprandial glucose” feature had the lowest correlation. In contrast, the “urine microalbumin/creatinine” feature is identified as the least important for both SVM and MLP. We removed these features, respectively, and retrained the model. The predicting results after removal are shown in **Table 9**. Compared with removing the “urine microalbumin/creatinine” feature, the prediction accuracy for several complication types improved (highlighted in the table) after removing the “postprandial glucose” feature. Notably, the accuracy for “Vascular complications of diabetes” improved from 94.83% to 95.17%, a 0.34% increase, followed by a 0.17% increase in the accuracy for “Complications of diabetes nephropathy”. The recall, F1, and AUC values also showed varying improvements and declines, but generally, the increases outweighed the decreases. Therefore, we believe that using the average feature importance can break the dependency of different models on certain features and is more suitable for reflecting the feature importance of ensemble learning models.

**Table 8:** The average importance of each feature.

Feature	Importance (XGBoost)	Importance (SVM)	Importance (RF)	Importance (MLP)	Average importance
creatinine	10.28%	1.04%	10.15%	3.76%	6.31%
cystatin C	10.39%	1.16%	9.95%	3.07%	6.15%
high-density lipoprotein cholesterol	10.01%	1.30%	8.74%	3.01%	5.76%
glycosylated hemoglobin	8.83%	1.39%	8.86%	3.55%	5.66%
24-hour urine protein	8.40%	0.88%	9.32%	2.04%	5.16%
urine microalbumin	7.19%	0.83%	7.81%	2.51%	4.90%
blood sugar	7.59%	1.08%	7.41%	3.32%	4.85%
uric acid	7.19%	0.83%	7.81%	2.51%	4.58%
urine microalbumin/creatinine	8.53%	0.37%	8.32%	1.09%	4.58%
total cholesterol	7.41%	0.82%	7.72%	2.13%	4.52%
low-density lipoprotein cholesterol	6.47%	0.89%	6.89%	2.83%	4.27%
postprandial glucose	6.58%	0.63%	6.19%	2.27%	3.92%

**Table 9:** Prediction results after removing features.

Complication Types	Classifiers	Accuracy	Recall	F1	AUC
After removing features “urine microalbumin/creatinine”					
No complications in diabetes	Stacking Classifier (logits) + Bayesian Optimization	86.17%	42.57%	50.89%	88.51%
Ocular complications of diabetes		95.83%	79.38%	86.03%	94.77%
Neurological complications of diabetes		92.50%	66.99%	75.41%	94.85%
Complications of diabetes nephropathy		98.00%	90.43%	93.41%	99.42%
Vascular complications of diabetes		94.50%	75.27%	80.92%	95.50%
Foot complications of diabetes		94.67%	76.79%	84.31%	94.49%
After removing features “postprandial glucose”					
No complications in diabetes	Stacking Classifier (logits) + Bayesian Optimization	**88.50%**	49.51%	59.17%	89.40%
Ocular complications of diabetes		**96.50%**	**82.47%**	**88.40%**	94.34%
Neurological complications of diabetes		93.17%	68.93%	77.60%	95.92%
Complications of diabetes nephropathy		**98.50%**	**93.62%**	**95.14%**	99.76%
Vascular complications of diabetes		**95.17%**	81.72%	**83.98%**	97.00%
Foot complications of diabetes		95.00%	78.57%	85.44%	**96.33%**
After removing features “postprandial glucose” and “low-density lipoprotein cholesterol”					
No complications in diabetes	Stacking Classifier (logits) + Bayesian Optimization	88.17%	45.54%	56.44%	88.58%
Ocular complications of diabetes		94.17%	71.13%	79.77%	94.73%
Neurological complications of diabetes		92.50%	66.02%	75.14%	94.61%
Complications of diabetes nephropathy		98.00%	90.43%	93.41%	**99.79%**
Vascular complications of diabetes		94.00%	79.57%	80.43%	96.24%
Foot complications of diabetes		95.50%	78.57%	86.70%	95.73%

Bold means the optimal values among different methods for the same evaluation metric.

Next, we removed two features to examine the model’s prediction accuracy. This experiment tried different combinations of removing two features and finally found that accuracies decreased mainly after removing “postprandial glucose” and “low-density lipoprotein cholesterol”. The prediction results are shown in **Table 9**. This reduction did not occur when only one irrelevant feature was removed. Therefore, removing two features did not consistently improve the model’s prediction accuracy while reducing costs.

Finally, for cost considerations, we obtained the cost of each sampling indicator from the hospital where the dataset was collected, as shown in **Table 10**. Our final model is combined with the experimental results above, stacking classifier with Bayesian optimization after removing the “postprandial glucose” feature. The cost of sampling all 12 features is 160 RMB, while this “postprandial glucose” indicator costs 4 RMB. Therefore, compared to sampling 12 indicators simultaneously, sampling only 11 features can reduce medical expenses by 2.5%, improving prediction accuracy.

**Table 10:** Cost of each feature.

Features	Cost(RMB)
uric acid	3
24-hour urine protein	6
high-density lipoprotein cholesterol	8
low-density lipoprotein cholesterol	4
total cholesterol	4
postprandial glucose	4
cystatin C	15
creatinine	4
microalbumin	24
microalbumin/creatinine	24
glycated hemoglobin	60
blood glucose	4
Total	160

## 4 Conclusion

In summary, this paper addresses the prediction of diabetic complications through laboratory medicine indicators. The aim is to provide diabetic patients with an early understanding of their potential complications following basic tests upon hospital admission. This approach provides a more accurate risk assessment for doctors and patients, potentially optimizing clinical decision-making. Initially, this study collected sample data from the laboratory department of a reputable hospital. Through data cleaning and label classification, a comprehensive data set was constructed, supporting this experiment’s training and prediction processes and facilitating further research in this field. Subsequently, by comparing the prediction results of Random Forest, XGBoost, SVM, MLP, Stacking Classifier, and Stacking Classifier with Bayesian Optimization, the experimental results indicate that the Stacking Classifier with Bayesian Optimization exhibits superior performance in predicting diabetic complications. Notably, the Stacking Classifier with Bayesian Optimization achieved an accuracy of 98.33% and an AUC of 99.71% in predicting diabetic nephropathy. Furthermore, to reduce patient testing costs, this paper analyzed the feature heat map and incrementally removed one and two features to observe changes in prediction accuracy. The experimental results demonstrate that after removing “postprandial glucose,” the overall prediction accuracy further improved, and the reduction in testing requirements led to a 2.5% decrease in the patient’s medical expenses.

## Data Availability

The datasets presented in this study can be found in online repositories. The names of the repository/repositories and accession number(s) can be found in the article/supplementary material.

## Footnotes

1
https://jsnydefy.com/index.php?c=category&id=2

2
https://www.gov.cn/zhengce/zhengceku/2023-02/28/content_5_743658*.htm*
